# Identification of IgE Cross-reactive Allergens Causing Food Allergies Using Murine Models

**DOI:** 10.14789/jmj.JMJ23-0045-R

**Published:** 2024-03-28

**Authors:** RISA YAMAMOTO, KUMI IZAWA, TOMOAKI ANDO, AYAKO KAITANI, AKIE MAEHARA, NOBUHIRO NAKANO, HIDEOKI OGAWA, KO OKUMURA, JIRO KITAURA

**Affiliations:** 1 Atopy (Allergy) Research Center, Juntendo University Graduate School of Medicine, Tokyo, Japan; 1 Atopy (Allergy) Research Center, Juntendo University Graduate School of Medicine, Tokyo, Japan; 2 Juntendo University School of Medicine (6^th^ year medical student), Tokyo, Japan; 2 Juntendo University School of Medicine (6^th^ year medical student), Tokyo, Japan

**Keywords:** food allergy, IgE cross-reactivity, house dust mite, salmon, murine model

## Background

Pollen food allergy syndrome (PFAS)/oral allergy syndrome (OAS) is caused by IgE cross-reactive allergens. Generally, PFAS has been diagnosed by taking a dietary history, measuring serum levels of allergen-specific IgE, and performing a skin prick test. However, the molecular mechanisms by which aeroallergens cross-react with food allergens are elusive. In this study, we aimed to develop methods for comprehensive identification of unknown IgE cross-reactive allergens, that may cause food allergies, using murine models.

## Methods

Mice were sensitized by intraperitoneal administration of either alum alone as a control or alum plus pollen (e.g., ragweed, birch) or Dermatophagoides pteronyssinus (Der p) extract. Allergenic protein microarray analysis was conducted using mouse serum to identify food extract highly bound to serum IgE from the sensitized mice. IgE cross- reactivity was evaluated by ELISA and murine models of local anaphylaxis. IgE cross-reactive food proteins were identified by mass spectrometry after protein separation. Recombinant proteins of interest were generated for further analysis.

## Results

Ragweed pollen showed strong IgE cross-reactivity with fennel and black pepper among edible plants both in vitro and in vivo ^1)^. IgE cross-reactivity was also observed between coho salmon and Der p. In addition, mass spectrometry analysis identified tropomyosin as the IgE cross-reactive protein contained in coho salmon and Der p extracts in our models ^2)^.

## Conclusions

**Figure 1:**
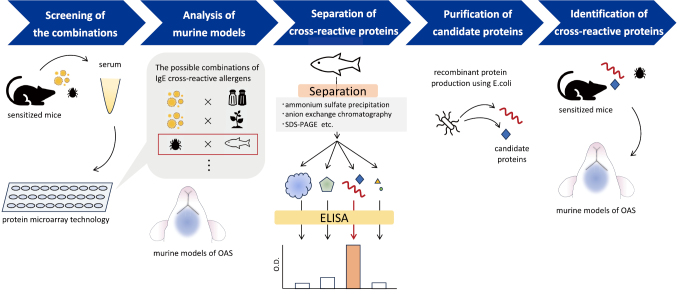
A new method to comprehensively identify IgE cross-reactive allergens

We developed a new screening method using allergenic protein microarray technology and murine model sensitized with environmental allergen. This method will be useful to identify the unknown IgE cross-reactive allergens that may be responsible for food allergies (Figure 1).

## Funding

This study was supported by JSPS KAKENHI Grant (Numbers 17H04217, 20H03721, 23H02946) and a Grant-in-Aid for Special Research in Subsidies for ordinary expenses of private schools from the Promotion and Mutual Aid Corporation for Private Schools of Japan.

## Author contributions

RY performed all the experiments and participated in writing the manuscript. KI assisted with the analysis of murine model and the in vitro experiments, analyzed the data, and actively participated in manuscript writing. TA assisted with the in vitro experiments and statistical analysis and analyzed the data. AM assisted with the in vitro experiments. AK and NN assisted with the in vivo experiments. HO and KO analyzed the data. JK conceived the project, analyzed the data, and actively participated in manuscript writing. All authors contributed to the article. All authors read and approved the final manuscript.

## Conflicts of interest statement

The authors declare that there are no conflicts of interest.
